# Out-of-maternity deliveries in France: A nationwide population-based study

**DOI:** 10.1371/journal.pone.0228785

**Published:** 2020-02-24

**Authors:** Evelyne Combier, Adrien Roussot, Jean-Louis Chabernaud, Jonathan Cottenet, Patrick Rozenberg, Catherine Quantin

**Affiliations:** 1 Biostatistics and Bioinformatics (DIM), Inserm, France University Hospital, Bourgogne Franche-Comté University, Dijon, France; 2 Neonatal and Pediatric Emergency Transport Team and NICU, Antoine-Beclere Hospital, AP-HP, Paris Saclay University, Clamart, France; 3 Versailles Saint-Quentin University, Department of Obstetrics and Gynecology, Poissy-Saint Germain Hospital, Poissy, France; 4 Biostatistics, Biomathematics, Pharmacoepidemiology and Infectious Diseases (B2PHI), INSERM, UVSQ, Institut Pasteur, Université Paris-Saclay, Paris, France; University of New South Wales, AUSTRALIA

## Abstract

**Introduction:**

In France, many maternity hospitals have been closed as a result of hospital restructuring in an effort to reduce costs through economies of scale. These closures have naturally increased the distance between home and the closest maternity ward for women throughout the country. However, studies have shown a positive correlation between this increase in distance and the incidence of unplanned out-of-maternity deliveries (OMD). This study was conducted to estimate the frequency of OMD in France, to identify the main risk factors and to assess their impact on maternal mortality and neonatal morbidity and mortality.

**Materials and methods:**

We conducted a population-based observational retrospective study using data from 2012 to 2014 obtained from the French hospital discharge database. We included 2,256,797 deliveries and 1,999,453 singleton newborns in mainland France, among which, 6,733 (3.0‰) were OMD. The adverse outcomes were maternal mortality in hospital or during transport, stillbirth, neonatal mortality, neonatal hospitalizations, and newborn hypothermia and polycythemia. The socio-residential environment was also included in the regression analysis. Maternal and newborn adverse outcomes associated with OMD were analyzed with Generalized Estimating Equations regressions.

**Results:**

The distance to the nearest maternity unit was the main factor for OMD. OMD were associated with maternal death (aRR 6.5 [1.6–26.3]) and all of the neonatal adverse outcomes: stillbirth (3.3 [2.8–3.8]), neonatal death (1.9 [1.2–3.1]), neonatal hospitalization (1.2 [1.1–1.3]), newborn hypothermia (5.9 [5.2–6.6]) and newborn polycythemia (4.8 [3.5–6.4]).

**Discussion:**

In France, OMD increased over the study period. OMD were associated with all the adverse outcomes studied for mothers and newborns. Caregivers, including emergency teams, need to be better prepared for the management these at-risk cases. Furthermore, the increase in adverse outcomes, and the additional generated costs, should be considered carefully by the relevant authorities before any decisions are made to close or merge existing maternity units.

## Introduction

In France, many maternity hospitals have been closed as a result of hospital restructuring in an effort to reduce costs through economies of scale. These closures have naturally increased the distance between home and the closest maternity ward for women throughout the country. However, studies have shown a positive correlation between this increase in distance and the incidence of unplanned out-of-maternity deliveries (OMD, also called out-of-hospital deliveries), which are hazardous events for both mother and child [1–4]. Because of their accidental nature and the frequent need for prompt medical attention, there is a potential risk of increased maternal and neonatal mortality and morbidity [5–7].

While the neonatal outcomes for OMD have been studied at length, there is little published data on adverse maternal outcomes. The few existing studies have focused on smaller geographic areas, and the resulting cohorts were limited in their ability to detect rare events such as maternal death.

In France in 2016, according to data from the French national statistical institute (Institut National de la Statistique et des Études Économiques [INSEE]), less than 1% of births occurred outside a maternity hospital (most often at home). They were most often planned to take place with assistance ("planned delivery") and only 0.1% took place without any assistance [8]. When a mobile emergency service was called, the medical team delivered the newborn in a third of cases [9].

Therefore a larger and, if possible, nationwide study is needed to provide better awareness of the burden and complications associated with OMD.

Our objectives were i) to estimate the frequency of out-of-maternity deliveries in France and identify the main risk factors, ii) to assess the impact of out-of-maternity deliveries on maternal mortality and on neonatal morbidity and mortality.

## Materials and methods

### Study design and setting

We conducted a nationwide population-based study of all women who gave birth at or after 24 weeks of gestation from 2012 to 2014 in mainland France.

### Selection of patients

The study data comprised all deliveries recorded from 2012 to 2014 in the French hospital database (Programme de médicalisation des systèmes d'information [PMSI]). The PMSI collects the discharge abstracts (DA) from all hospitals in France and is 100% exhaustive for in-hospital deliveries [10]. The data included maternal age, gestational age (GA), length of stay, and hospital death. Diagnoses and procedures are coded according to the International Classification of Diseases (ICD-10) and to the French Classification of Medical Procedures (CCMP). The geographic scale used for this analysis was the geographic code of residence (zip codes) recorded in the PMSI.

We studied all deliveries in France at or after 24 weeks of gestation (WG). They were identified from the ICD-10 codes Z37 (“Outcome of delivery”) and the delivery procedure codes. OMD were identified when code Z3900 (“Care and examination immediately after delivery out-of-health hospital”) was the main diagnosis in the DA following delivery. Liveborn singleton DAs were identified from codes Z38.0 (“Single liveborn infant, born in hospital”) and Z38.1 (“Single liveborn infant, born outside hospital”) and age in days equal to zero.

**Fig 1:**
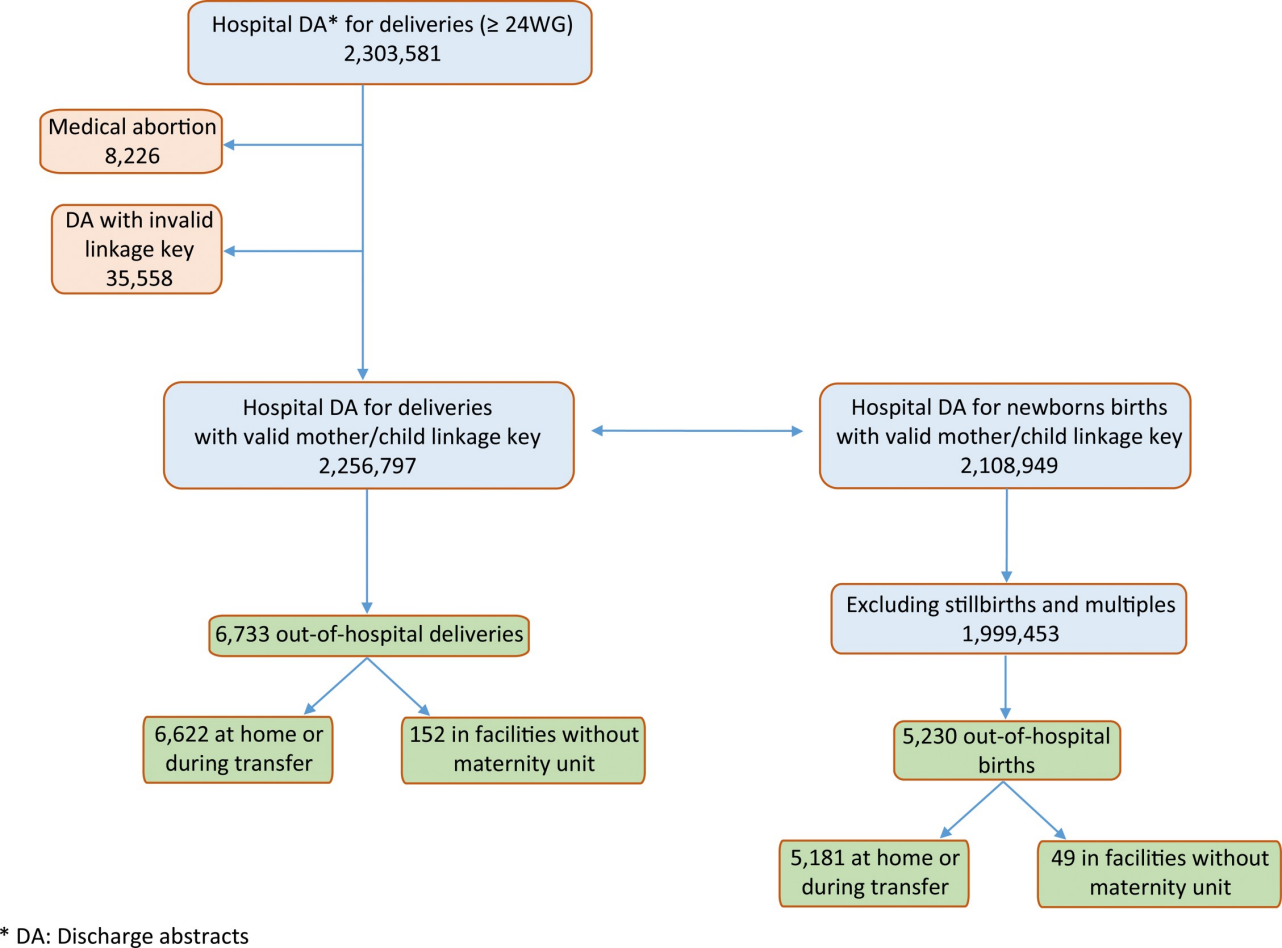
Flow chart of study population for the period 2012–2014.

The PMSI database allows the linkage of DAs for consecutive hospitalizations and the linkage of mothers’ and children’s DAs for singleton pregnancy thanks to a common identifier used for both (**Fig 1**), in the framework of the secure anonymized information linkage in use since April 2012. Our epidemiologic follow-up included all PMSI data from the beginning of pregnancy until 42 days after delivery for women and from the first 28 days for infants. Women who were hospitalized more than 24 hours before delivery were identified from the time between hospital admission and delivery.

We excluded terminations of pregnancy for medical reasons, DAs without zip code (0.1%) and DAs with a zip code corresponding to an overseas territory or a foreign country, because the distance from home to the maternity unit could not be calculated. After these exclusions, our study retained 2,256,797 deliveries and 1,999,453 singleton live newborns.

### Variables of interest

#### Individual

The variables of interest were maternal age (<25, 25–39, ≥40 years), prematurity (<37 WG, ≥37 WG) and high-risk pregnancy when one of the following ICD-10 codes was recorded for antepartum hospitalization or delivery DA: O10-O16 (“Edema, proteinuria and hypertensive disorders in pregnancy, childbirth and the puerperium”), O24 (“Diabetes mellitus in pregnancy“), O99 (“Other maternal diseases classifiable elsewhere but complicating pregnancy, childbirth and the puerperium“), Z35 (“Supervision of high-risk pregnancy“), and C00-D48 (“Neoplasms“). In our data, placenta previa and all other conditions influencing pregnancy are included in the ICD-10 O99 code.

#### Socio-residential environment

*Distance between the mother's home and the closest maternity ward*. We calculated the minimum distance by road between each maternal zip code and the nearest maternity unit.

*Material and social deprivation index*. Based on French population and household income census data, we calculated the average rates of unemployment, industrial workers, immigrants, people without a high school diploma or with a post-secondary qualification and the rate of non-taxable households for each zip code. These data were correlated and pooled in a material and social deprivation index according to the bi-dimensional scale put forward by Pampalon [11]. Five levels of material and social deprivation were defined: level 1 (the least materially and socially disadvantaged population clusters), level 2 (national average for each variable), level 3 (social deprivation only), level 4 (material deprivation only), level 5 (social and material deprivation, the most disadvantaged geographic codes).

*Levels of urbanization*. We grouped the zip codes into three categories of urbanization and number of jobs (using geographic data produced by the French national statistical institute [INSEE]): 1) the largest urban areas (≥10 000 jobs), 2) their surrounding areas (suburban areas), and 3) other urban, suburban and rural areas.

#### Outcomes

We studied the main adverse outcomes after an OMD available in the PMSI data: maternal mortality in hospital or during transport, stillbirth, neonatal mortality, neonatal hospitalization, newborn hypothermia and newborn polycythemia.

Maternal deaths were identified at discharge with vital status upon discharge from hospital or an ICD-10 code O95 (“Obstetric death of unspecified cause”) by women who delivered until 42 days after delivery. The time of death was calculated from the date on which the act of delivery was recorded. Stillbirths and neonatal deaths were identified from diagnosis-related groups, or from the vital status upon discharge from hospital.

Neonatal hospitalization was recorded when the first discharge abstract included a transfer to another nursing unit or from one hospital to another, a corresponding payment of surcharge rates, or a diagnosis-related group code 15M02Z (“Early neonatal transfer*”*).

Hypothermia and polycythemia were identified on birth DAs from neonatal hospitalizations with ICD-10 codes P80 ("Hypothermia of newborn”), P611 (“Polycythemia neonatorum”) or P583 (“Neonatal jaundice due to polycythemia”), respectively.

### Statistical analysis

Qualitative variables were expressed as percentages and compared using Pearson's Chi-squared test or Fisher’s exact test. We used a Somers’d test to evaluate trends over the years. The multivariate analyses were done using regressions based on Generalized Estimating Equations (GEE_s_) with a log link function and negative binomial distribution to take into account the data correlations *(zip codes and years)* and overdispersion. The two contextual variables (deprivation index and levels of urbanization) were tested one by one in the regressions, but the model converged better with both, according to the QIC statistic.

Risk factors for maternal mortality and stillbirth were analyzed for all OMD. Adverse neonatal outcomes were only analyzed for births before arrival (BBA). For neonatal adverse outcomes, we performed two sensitivity analyses by including the 9.6% of DAs for women who could not be linked to the DAs of their babies in the database. We used at first a multiple imputation (MI) method [12], according to the repartition of adverse outcomes identified in observed data, i.e. using the distribution of the observed data to estimate a set of plausible values for the missing data. Secondly, for all of the missing newborns (in and out-of-maternity born), we considered that there was no adverse outcome (maximum bias) and we used the information on the covariates of the mothers’ DA. The results of the two sensitivity analyses are presented in **S1 Table**.

Statistical analyses were performed using SAS^®^ version 9.4 (SAS institute Inc., Cary, NC, USA). Statistical significance was defined as a *P*-value <0.05.

Distances were calculated using CHRONOMAP^©^ for MAPINFO^©^ software and IGN ROUTE 500^®^ digital road network.

### Details of ethics approval

The national hospital database was transmitted by the national agency for the management of hospitalization data (ATIH number 2015-111111-47-33). The French Committee for Data Protection approved this study (Commission Nationale de l'Informatique et des Libertés, registration number 1576793). This study was conducted in accordance with the Declaration of Helsinki. Individual written consent was not needed for this study.

## Results

### Characteristics of the population

The characteristics of the mothers, pregnancies and newborns are presented in **Table 1**. From 2012 through 2014, we identified 2,256,797 deliveries at or after 24 WG in mainland France, from the French hospital database. Multiple pregnancies accounted for 1.61% (36,118) of all deliveries.

**Table 1:** Characteristics of mothers, pregnancies and newborns: Change over time.

Characteristics	2012	2013	2014	3 YEARS	Somers’d
	No. (%)	No. (%)	No. (%)	No. (%)	*P*<|Z|
Mothers and pregnancies	762,726	752,793	741,278	2,256,797	
Antenatal hospitalizations					
Delivery>24hours of maternal admission	300,122 (39.4)	302,715 (40.2)	298,535 (40.3)	901,372 (39.9)	<0.001
Delivery>48hours of maternal admission	59,340 (7.8)	61,105 (8.1)	58,880 (7.9)	179,325 (8.0)	<0.001
24hours<Hospitalizations<48hours	240,782 (31.6)	241,610 (32.1)	239,662 (33.6)	722,075 (32.0)	<0.001
Out-of-hospital deliveries					
All deliveries	2,104 (2.8)*	2,300 (3.1)*	2,329 (3.1)*	6,733 (3.0)*	<0.001
Delivery<24hours of maternal admission	2,104 (4.5)*	2,300 (5.1)*	2,329 (5.3)*	6,733 (5.0)*	<0.001
Babies born before arrival	2,074 (2.7)*	2,253 (3.0)*	2,295 (3.1)*	6,622 (2.9)*	< .0001
In hospital but out-of-maternity deliveries	30 (0.04)*	47 (0.06)*	34 (0.05)*	111 (0.05)*	0.5207
Multiple pregnancies	10,700 (1.4)	12,939 (1.7)	12,479 (1.7)	36,118 (1.6)	<0.001
Deliveries with stillbirth	2,949 (3.9)*	2,896 (3.8)*	2,668 (3.6)*	8,513 (3.8)*	0.008
Maternal death (< = D42)	35 (4.6) ^†^	31 (4.1) ^†^	32 (4.3) ^†^	98 (4.3) ^†^	0.7993
Gestational age at delivery (weeks)					
24–32	9,935 (1.3)	9,573 (1.3)	8,630 (1.2)	28,138 (1.3)	<0.001
33–34	9,527 (1.3)	9,494 (1.3)	8,637 (1.2)	27,658 (1.2)	
35–36	30,211 (4.0)	29,351 (3.9)	28,611 (3.9)	88,173 (3.9)	
37–44	713,053 (93.5)	704,375 (93.6)	695,400 (93.8)	2,112,828 (93.6)	
Maternal age (years)					
<25	125,575 (16.5)	119,271 (15.8)	112,168 (15.1)	357,014 (15.8)	<0.001
25–39	608,428 (79.8)	603,515 (80.2)	598,866 (80.8)	1,810,809 (80.2)	
40+	28,723 (3.8)	30,007 (4.0)	30,244 (4.1)	88,974 (3.9)	
High-risk pregnancy ^a^	99,892 (13.1)	101,972 (13.6)	102,661 (13.9)	304,525 (13.5)	<0.001
Distance to the closest maternity unit (km)					
0–15	557,274 (73.1)	553,827 (73.6)	548,487 (74.0)	1,659,588 (73.5)	<0.001
16–30	172,153 (22.6)	165,857 (22.0)	161,001 (21.7)	499,011 (22.1)	
31–45	28,775 (3.8)	28,998 (3.9)	27,543 (3.7)	85,316 (3.8)	
46–90	4,524 (0.6)	4,111 (0.6)	4,247 (0.6)	12,882 (0.6)	
Material and social deprivation index					
No deprivation: Level 1	68,908 (9.0)	68,380 (9.1)	68,980 (9.3)	206,268 (9.1)	0.378
Middle class: Level 2	491,938 (64.5)	485,315 (64.5)	475,226 (64.1)	1,452,479 (64.4)	
Material deprivation: Level 3	24,623 (3.2)	22,978 (3.1)	23,382 (3.2)	70,983 (3.2)	
Social deprivation: Level 4	112,489 (14.8)	111,426 (14.8)	110,287 (14.9)	334,202 (14.8)	
Material and social deprivation: Level 5	64,768 (8.5)	64,694 (8.6)	63,403 (8.6)	192,865 (8.6)	
Level of urbanization					
Major urban centers	491,035 (64.4)	488,277 (64.9)	484,693 (65.4)	1,464,005 (64.9)	<0.001
Surrounding suburbs	161,817 (21.2)	158,761 (21.1)	154,502 (20.8)	475,080 (21.1)	
Other areas	109,874 (14.4)	105,755 (14.1)	102,083 (13.8)	317,712 (14.1)	
**Single live births**	653,014	672,604	673,835	1,999,453	
Babies born before arrival	1,560((2.4)*	1,733 (2.6)*	1,888(2.8)*	5,181 (2.6)*	<0.001
Male	334,276(51.2)	344,564 (51.2)	343,987(51.1)	1,022,827 (51.2)	0.1010
Malformations, and chromosomal abnormalities ^b^	40,623(6.2)	41,770 (6.2)	39,890(5.9)	122,283 (6.1)	<0.001
Neonatal death(< = D27)	935(1.4)*	979 (1.5)*	838(1.2)*	2,752 (1.4)*	0.003
Polycythemia	1,129(0.2)	1,215 (0.2)	1,143(0.2)	3,487 (0.2)	0.635
Neonatal hemorrhage	1,746(0.3)	1,862 (0.3)	1,788(0.3)	5,396 (0.3)	0.806
Hypothermia	6,768(1.0)	7,204 (1.1)	6,794(1.0)	20,766 (1.0)	0.098
Newborn hospitalization	57,851 (8.9)	59,016 (8.8)	56,321 (8.4)	173,188 (8.7)	<0.001

* per 1,000

^†^ per 100,000

^a^ ICD-10: O10-O16: Edema, proteinuria and hypertensive disorders in pregnancy, childbirth and the puerperium, O24: Diabetes mellitus in pregnancy, O99: Other maternal diseases, Z35: Supervision of high-risk pregnancy, C00-D48: Neoplasms

^b^ Congenital malformations, and chromosomal abnormalities (ICD-10: Q00-Q99), including all minor anomalies

From 2012 to 2014, 6,733 (3.0‰) deliveries took place out-of-maternity, including unexpected out-of-maternity deliveries and home deliveries requiring transfer to a hospital. For these deliveries, we counted 6,622 babies born before arrival and 111 deliveries that occurred in a hospital but not in a maternity unit, 41 of which occurred in maternity units that had closed and were replaced by antenatal consultations centers (*Centre Périnatal de Proximité*, *CPP*).

Over the study period, we identified a significant increase (P < .001) in out-of-maternity delivery rates over time, from 2.8‰ (2012) to 3.1‰ (2014). Antenatal hospitalization rates also increased (39.9% overall, P < .001), for both short-term hospitalizations (24h>delivery>48h; 32.0%, P < .001) and longer hospital stays (deliveries>48h after admission; 8.0%, P < .001). From 2012 to 2014, more women were living in major urban centers (64.4% in 2012–65.4% in 2014, P < .001), resulting in more women living less than 16 km from a maternity unit (73.1% in 2012–74.0% in 2014, P < .001), while the rate of women living at more than 30 km remained unchanged (4.4%). No women lived further than 90 km away from a maternity hospital. An analysis of the socio-residential environment showed that 9.1% of women lived in an area classified level 1 on the material and social deprivation index (least disadvantaged) and 8.6% lived in a level 5 area (most disadvantaged), with no change over the period.

### Main results

The majority of women in the OMD group were aged 25 to 39 years (78.8%) **(Table 2)**, gave birth at 37 WG or more (90.5%) and had an uncomplicated pregnancy (87.2%). In addition, they lived mainly in major urban centers or their suburbs (77.2%), in areas ranked level 2 (middle class) of the deprivation index (62.0%) and less than 16 km from a maternity hospital (62.6%).

**Table 2:** Comparison of characteristics and adverse outcomes according to the place of delivery.

	Maternity unit deliveries	Out-of- hospital deliveries	*P*	Crude relative risk (95%CI)
	No. (^0^/_0_)	No. (^0^/_0_)		
**Characteristics**				
**All deliveries**	2,250,064 (100.0)	6,733 (100.0)		
Maternal age (years)				
<25	355,964 (15.8)	1,050 (15.6)	*<0*.*001*	1.0 (0.9–1.1)
25–39	1,805,502 (80.3)	5,307 (78.8)		Reference
40+	88,598 (3.9)	376 (5.6)		1.5 (1.4–1.7)
Prematurity: gestational age<37 weeks	143,329 (6.4)	640 (9.5)	*<0*.*001*	1.5 (1.4–1.7)
Congenital malformations and chromosomal abnormalities*	121,900 (6.1)	337 (6.4)	*0*.*319*	1.1 (0.9–1.2)
High risk pregnancy	303,663 (13.5)	862 (12.8)	*0*.*058*	0.9 (0.9–1.0)
Sex of newborn (*male*)	102,0404 (51.2)	2,423 (46.3)	*<0*.*001*	0.8 (0.8–0.9)
Distance to the closest maternity unit (km)				
0–15	1,655,372 (73.6)	4,216 (62.6)	*<0*.*001*^†^	Reference
16–30	497,182 (22.1)	1,829 (27.2)		1.5 (1.4–1.5)
31–45	84,755 (3.8)	561 (8.3)		2.6 (2.4–2.9)
46–90	12,755 (0.6)	127 (1.6)		3.9 (3.2–4.8)
Material and social deprivation index				
No deprivation: level 1	205,879 (9.2)	385 (5.8)	*<0*.*001*	0.6 (0.6–0.7)
Middle class: level 2	1,448,301 (64.4)	4,178 (62.0)		Reference
Material deprivation only: level 3	70,641 (3.1)	342 (5.1)		1.7 (1.5–1.9)
Social deprivation only: level 4	333,082 (14.8)	1,120 (16.6)		1.2 (1.1–1.3)
Material and social deprivation: level 5	192,161 (8.5)	704 (10.5)		1.3 (1.2–1.4)
Level of urbanization				
Major urban centers	1,460,172 (64.9)	3,833 (56.9)	*<0*.*001*	0.5 (0.5–0.6)
Surrounding suburbs	473,718 (21.1)	1,362 (20.3)		0.6 (0.6–0.6)
Other areas	316,174 (14.1)	1,538 (22.8)		Reference
**Adverse outcomes**				
Maternal death D_0_-D_42_	94 (4.2) ^‡^	4 (59.4) ^‡^	*<0*.*001*	13.7 (5.2–35.7)
Delivery with stillbirth	8,380 (3.7)^§^	133 (19.8) ^§^	*<0*.*001*	5.2 (4.4–6.2)
**All live births**	1,994,272 (100.0)	5,181 (100.0)^¶^		
Neonatal death D0-D27	2,736 (1.4) ^||^	16 (3.1) ^||^	*0*.*003*	2.4 (1.5–3.8)
Neonatal Hospitalization	172,605 (8.7)	583 (11.3)	*<0*.*001*	1.4 (1.3–1.5)
Hypothermia of newborn	20,445 (1.0)	321 (6.2)	*<0*.*001*	6.3 (5.6–7.0)
Neonatal Polycythemia	3,443 (0.2)	44 (0.9)	*<0*.*001*	4.9 (3.7–6.6)

* Congenital malformations, and chromosomal abnormalities (ICD-10: Q00-Q99)

^†^ *Somers’d*: *p<|Z|*

^‡^ per 100,000 deliveries

^§^ per 1,000 deliveries

^||^ per 1,000 live births

^¶^ Babies born before arrival

The women who delivered out-of-maternity did not have more high-risk pregnancies (cRR 0.9, 95% CI 0.9–1.0), but they had a higher risk of delivering before 37 WG (cRR 1.5, 95% CI 1.4–1.7). Furthermore, older women were more likely to deliver out-of-maternity (for 40 and older, crude relative risk [cRR] 1.5, 95% CI 1.4–1.7). The cRR of OMD increased significantly with the distance to the closest maternity unit from 1.5 (95% CI 1.4–1.5) at 16–30 km, to 3.9 (95% CI 3.2–4.8) at more than 45 km away. Compared with the maternity-unit delivery group, more women with OMD lived in disadvantaged areas (levels 3 to 5 of the deprivation index) and fewer of them lived in major urban centers or their surrounding suburbs. These differences were significant (P < .001). From 2012 to 2014, there were 98 maternal deaths, including 4 women in the OMD group. The crude risk was 4.2/100,000 deliveries in maternity vs 59.4 /100,000 deliveries in OMD group (cRR 13.7, 95% CI 5.2–35.7). Three of these four deaths were from obstetric causes: two hemorrhages and one amniotic fluid embolism. The fourth maternal death was not for obstetric reasons. We also observed a significant increase for all adverse outcomes in newborns from the OMD group.

For all deliveries and after adjustment **(Table 3)**, the covariates remained significant except for areas defined as level 3 of the deprivation index. The same covariates were significant for women who gave birth at the maternity less than 24 hours after admission (Table 2: N = 1,355,425). In this group, the risk of OMD increased significantly with the distance to the closest maternity, as shown by the adjusted relative risks (aRR): 1.5 (95% CI 1.4–1.6) for 16–30 km, 2.3 (95% CI 2.1–2.6) for 30–45 km and 3.6 (95% CI 2.9–4.4) for 46 km or more. The risk of OMD decreased for those living in major urban centers (aRR 0.9, 95% CI 0.8–0.9), in their surrounding suburbs (aRR 0.8, 95% CI 0.7–0.9) and for level 1 of the deprivation index (aRR 0.7, 95% CI 0.6–0.8). Conversely, the risk increased for levels 4 and 5 of the deprivation index ([aRR 1.2, 95% CI 1.1–1.3] and [aRR1.3, 95% CI 1.2–1.5], respectively). Giving birth prematurely (aRR 2.2, 95% CI 2.1–2.4) or being aged 40 and over (aRR 1.6, 95% CI 1.4–1.8) increased individual risk. On the other hand, the aRR was not significant for high-risk pregnancies (1.1, 95% CI 0.99–1.2). There was no significant interaction between individual and environmental variables.

**Table 3:** Risks (^0^/_00_) and adjusted relative risks of out-of-maternity deliveries.

** **	All deliveries 2012–2014				Deliveries with no antenatal hospitalization*			
	N = 2,256,797				N = 1,355,425			
	All deliveries	Out-of-maternity deliveries			All deliveries	Out-of-maternity deliveries		
		No. (^0^/_00_)	*P*	Adjusted Relative risk (95%CI)		No. (^0^/_00_)	*P*	Adjusted Relative risk (95%CI)
**Distance to the closest maternity unit (km)**								
0–15	1,659,588	4,216 (2.5)	<0.001^†^	Reference	997,866	4,216 (4.2)	<0.001^†^	Reference
16–30	499,011	1,829 (3.7)		1.4 (1.3–1.6)	299,806	1,829 (6.1)		1.5 (1.4–1.6)
30–45	85,316	561 (6.6)		2.3 (2.0–2.6)	50,597	561 (11.1)		2.3 (2.1–2.6)
46–90	12,882	127 (9.9)		3.2 (2.6–4.0)	7156	127 (17.7)		3.6 (2.9–4.4)
**Maternal age (years)**								
< 25	357,014	1,050 (2.9)	0.001	0.9 (0.9–1.0)	205,190	1,050 (5.1)	0.0138	1.0 (0.9–1.1)
25–39	1,810,809	5,307 (2.9)		Reference	1,100,903	5,307 (4.8)		Reference
40+	88,974	376 (4.2)		1.5 (1.3–1.6)	49,332	376 (7.6)		1.6 (1.4–1.8)
**Gestational age at delivery (weeks)**								
37+	2,112,828	6,093 (2.9)	<0.001	Reference	1,295,262	6,093 (4.7)	<0.001	Reference
24–36	143,969	640 (4.4)		1.5 (1.4–1.7)	60,163	640 (10.6)		2.2 (2.1–2.4)
**High-risk pregnancy**								
No	1,952,272	5,871 (3.0)	NS	Reference	1,193,418	5,781 (4.9)	NS	Reference
Yes	304,525	862 (2.8)		0.9 (0.9–1.01)	162,007	862 (5.3)		1.1 (0.99–1.2)
**Material and social deprivation index**								
No deprivation: level 1	206,268	389 (1.9)	<0.001	0.7 (0.7–0.8)	129,505	389 (3.0)	<0.001	0.7 (0.6–0.8)
Middle class: level 2	1,452,479	4,178 (2.9)		Reference	869,732	4,178 (4.8)		Reference
Material deprivation only: level 3	70,983	342 (4.8)		1.1 (0.9–1.2)	40,892	342 (8.4)		1.1 (1.0–1.3)
Social deprivation only: level 4	334,202	1,120 (3.4)		1.2 (1.1–1.3)	202,698	1,120 (5.5)		1.2(1.2–1.3)
Material and social deprivation: level 5	192,865	704 (3.7)		1.3 (1.2–1.5)	112,598	704 (6.3)		1.3 (1.2–1.5)
**Level of urbanization**								
Major urban centers	1,464,005	3,833 (2.6)	<0.001	0.9 (0.8–0.95)	878,513	3,833 (4.4)	<0.001	0.9 (0.8–0.9)
Surrounding suburbs	475,080	1,362 (2.9)		0.8 (0.7–0.9)	287,671	1,362 (4.7)		0.8 (0.7–0.9)
Other areas	317,712	1538 (4.8)		Reference	189,241	1538 (8.1)		Reference

Out-of-maternity unit deliveries baseline rate (0/00): All deliveries 2.7 (95% CI 2.4–2.9)—Deliveries with no antenatal hospitalization 4.5 (95% CI 4.0–5.0)

* Deliveries < 24 hours after maternal admissions

^†^ Somers’d

The risk factors for maternal antenatal hospitalization are shown in **Table 4**. After adjustment, the risk was significantly higher for women living more than 46 km from a maternity unit (aRR 1.10, 95% CI 1.07–1.14). This risk also increased for women living in neighborhoods identified as level 3 or 5 of the deprivation index (aRR 1.04, 95% CI 1.03–1.06 and aRR 1.02, 95% CI 1.01–1.03, respectively). On the contrary, women living in neighborhoods classified level 1 were less often hospitalized (aRR 0.94, 95% CI 0.93–0.95). The level of urbanization had no influence. The risk of antenatal hospitalization was higher for women under 25 (aRR 1.07, 95% CI 1.07–1.08) or 40 and older (aRR 1.12, 95% CI 1.11–1.13). Hospitalization was also more frequent for women who delivered before 37 WG (aRR 1.48, 95% CI 1.47–1.49) and for high-risk pregnancies (aRR 1.18, 95% CI 1.18–1.9).

**Table 4:** Risks (^0^/_0_) and adjusted relative risks of antenatal hospitalization.

All deliveries 2012–2014 N = 2 256 797	All deliveries	Hospitalization: Delivery>1 day of maternal admission N = 901,372 (Risk/100 deliveries)		
		No. (‰)	P	Adjusted relative risk (95%CI)
Distance to the closest maternity unit (km)				
0–15	1,659,588	661,722 (39.9)	<0.001*	Reference
16–30	499,011	199,205 (39.9)		1.00 (0.99–1.01)
30–45	85,316	34,719 (40.7)		1.01 (0.99–1.03
46–90	12,882	5,726 (44.5)		1.10 (1.07–1.14)
Maternal age (years)				
< 25	357,014	151,824 (42.5)	<0.001	1.07 (1.07–1.08)
25–39	1,810,809	709,906 (31.5)		Reference
40+	88,974	39,642 (44.6)		1.12 (1.11–1.13)
Gestational age at delivery (weeks)				
37+	2,112,828	817,566 (38.7)	<0.001	Reference
24–36	143,969	83,806 (58.2)		1.48 (1.47–1.49)
High-risk pregnancy				
No	1,952,272	758,854 (38.9)	<0.001	Reference
Yes	304,525	142,518 (46.8)		1.18 (1.18–1.9)
Material and social deprivation index				
No deprivation/ level 1	206,268	76,763 (37.2)	<0.001	0.94 (0.93–0.95)
Middle class: level 2	145,2479	582,747 (40.1)		Reference
Material deprivation only: level 3	70,983	30,091 (42.4)		1.04 (1.03–1.06)
Social deprivation only: level 4	334,202	131,504 (39.4)		0.99 (0.97–0.99)
Material and social deprivation level: 5	192,865	80,267 (41.6)		1.02 (1.01–1.03)
Level of urbanization				
Major urban centers	1,464,005	585,492 (40.0)	<0.001	1.00 (0.99–1.01)
Surrounding suburbs	475,08	187,409 (39.5)		1.00 (0.99–1.02)
Other areas	317,712	128,471 (40.4)		Reference

* Somers’d

After adjustment for covariates (**Table 5**), OMD were associated with maternal death (aRR 6.5, 95% CI 1.6–26.3) and stillbirth (aRR 3.3, 95% CI 2.8–3.8), neonatal death (aRR 1.9, 95% CI 1.2–3.1), neonatal hospitalization (aRR 1.2, 95% CI 1.1–1.3), hypothermia (aRR 5.9, 95% CI 5.2–6.6) and polycythemia (aRR 4.8, 95% CI 3.5–6.4). Apart from neonatal hospitalization, distance to the nearest maternity hospital did not influence adverse outcomes. The risks of maternal death, stillbirth and neonatal death were increased for levels 4 and 5 of the deprivation index (vs. level 2). Advanced maternal age (≥ 40 years), preterm delivery (<37 WG) and high-risk pregnancy were significantly associated with all adverse outcomes for both women and newborns. From the results of the two sensitivity analyses (**S1 Table**), OMD remained associated with all adverse outcomes for women and newborns. The adjusted relative risks were lower but remained significant.

**Table 5:** Risk factors for adverse outcomes: Adjusted relative risk.

	All deliveries 2012–2014 N = 2,256,797 *(Out-of-maternity deliveries)*		Single babies born alive N = 1,999,453 (*Babies born before arrival*)			
	Maternal Death (D0-D42)	Delivery with stillborn	Neonatal Death (D0-D27)	Neonatal Hospitalization	Newborn Hypothermia	Neonatal Polycythemia
Unexpected out -of-hospital deliveries						
No	Reference	Reference	Reference	Reference	Reference	Reference
Yes	6.5 (1.6–26.3)	3.3 (2.8–3.8)	1.9 (1.2–3.1)	1.2 (1.1–1.3)	5.9 (5.2–6.6)	4.8 (3.5–6.4)
Distance to the closest maternity unit (km)						
0–15	Reference	Reference	Reference	Reference	Reference	Reference
16–30	0.7 (0.4–15)	1.0 (0.9–1.0)	1.0 (0.9–1.1)	1.1 (1.0–1.2)	1.0 (0.9–1.2)	1.0 (0.8–1.2)
31–45	0.5 (0.1–2.5)	1.0 (0.9–1.1)	1.2 (0.9–1.5)	1.2 (1.1–1.4)	1.1 (0.9–1.4)	1.0 (0.8–1.3)
46–90	1.7 (0.2–13.8)	0.9 (0.7–1.2)	1.2 (0.7–1.9)	1.0 (0.9–1.1)	0.9 (0.6–1.4)	1.0 (0.6–1.6)
Maternal age (years)						
<25	0.6 (0.3–1.1)	1.1 (1.0–1.1)	1.2 (1.1–1.3)	1.0 (1.0–1.1)	1.0 (0.9–1.1)	1.0 (1.0–1.1)
25–39	Reference	Reference	Reference	Reference	Reference	Reference
40+	2.8 (1.6–5.0)	1.4 (1.3–1.5)	1.4 (1.1–1.6)	1.2 (1.2–1.2)	1.1 (1.0–1.1)	1.4 (1.2–1.6)
Sex of newborn						
male			Reference	Reference	Reference	Reference
female			0.9 (0.8–0.9)	0.9 (0.9–0.9)	1.1 (1.0–1.1)	0.9 (0.9–1.0)
Gestational age at delivery (weeks)						
37+	Reference	Reference	Reference	Reference	Reference	Reference
24–36	4.2 (2.6–6.8)	34.1 (32.5–35.7)	30.9 (28.4–33.5)	9.2 (8.9–9.5)	2.1 (1.9–2.3)	4.2 (3.8–4.6)
High-risk pregnancy						
No	Reference	Reference	Reference	Reference	Reference	Reference
Yes	2.2 (1.4–3.5)	1.0 (1.0–1.1)	1.4 (1.3–1.6)	2.0 (2.0–2.1)	1.7 (1.7–1.8)	2.2 (2.0–2.4)
Material and social deprivation index						
No deprivation: level 1	0.8 (0.3–1.8)	0.9 (0.8–1.0)	0.8 (0.7–0.9)	1.0 (0.9–1.1)	1.1 (0.9–1.3)	0.8 (0.7–0.9)
Middle class: level 2	Reference	Reference	Reference	Reference	Reference	Reference
Material deprivation only: level 3	0.4 (0.1–3.2)	1.1 (1.0–1.3)	0.8 (0.7–1.1)	0.9 (0.8–0.9)	0.9 (0.7–1.1)	1.0 (0.8–1.2)
Social deprivation only: level 4	1.7 (1.0–2.9)	1.2 (1.2–1.3)	1.2 (1.0–1.3)	1.0 (1.0–1.1)	1.3 (1.1–1.6)	1.2 (1.1–1.5)
Material and social deprivation: level 5	2.1 (1.2–3.8)	1.3 (1.2–1.4)	1.2 (1.1–1.4)	0.9 (0.9–1.0)	0.9 (0.7–1.1)	0.7 (0.6–0.9)
Level of urbanization						
Major urban centers	0.9 (0.4–1.9)	1.1 (1.0–1.2)	1.1 (0.9–1.2)	1.1 (1.1–1.2)	0.9 (0.8–1.1)	0.9 (0.8–1.1)
Surrounding suburbs	0.8 (0.3–1.7)	1.0 (0.9–1.1)	1.0 (0.8–1.1)	1.0 (1.0–1.1)	0.9 (0.7–1.1)	0.8 (0.7–1.0)
Other areas	Reference	Reference	Reference	Reference	Reference	Reference

## Discussion

We identified 6,733 out-of-maternity deliveries (OMD) and 6,622 babies born before arrival (BBA) in France from 2012 to 2014. The risk of OMD was 3.0‰ for all deliveries, 5.0‰ for non-hospitalized women and 2.9‰ for BBA. These risks increased over the period, like in other countries [4,13,14] where the questions of the reorganization of perinatal care and increasing travel times have already been examined [15–17]. In France, for example, changes have resulted from a sharp drop in the number of maternity units, which went from 815 in 1996 to only 416 in 2016 [18].

Our results showed that OMD most commonly occur in women living less than 16 km from the nearest maternity ward, who deliver at term and without any risk factors. However, our study, like others, points to an increase in distance to the nearest maternity ward (expressed in kilometers or in minutes) as the most important risk factor for OMD. We found that the risk was multiplied by 1.4 when the distance went from 16–30 km to < 16 km, by 2.3 when the distance went to 31–45 km and 3.2 when the distance went to 46 km or more. This increase in risk is similar to what was recorded in France in 2005–2006 [19] and the gradient is consistent with the results of a Norwegian survey [20].

We were not able to statistically test the existence of a causal link between the increase in the rate of OMD during the investigation period and the closure of maternity wards. We would have needed finer scale data than what is available in the PMSI (for example, the municipality of residence) and the exact date of closure of the maternity hospitals, which is difficult to access [21].

However, there is indirect evidence of this relationship, such as the fact that 41 women gave birth in an institution whose maternity hospital was closed and replaced by an outpatient antenatal consultation center (*Centre Périnatal de Proximité*, CPP), increasing the distance to the nearest maternity hospital in the catchment area of these institutions. This is particularly true in the Burgundy region, which has been heavily affected by the closure of maternity hospitals [18,22,23], and has seen a significant increase in access times in rural areas, from less than 15 minutes up to one hour in some municipalities [24]. From 2000 to 2010, remote areas in Burgundy saw the closure of four maternity hospitals which were replaced by CPPs [17]. In Burgundy, eleven deliveries were recorded in three of these facilities during the three years of our study. These 11 deliveries represent 27% of the 41 CPPs deliveries recorded in the entire metropolitan area, while deliveries in Burgundy represented only 2.2% of the total in mainland France (49,910/2,256,797). Moreover, the rate of OMD (3.5‰) in Burgundy was higher than that recorded for the entire metropolitan area, which was only 3.0‰ (**Table 1**).

In parallel with the increase of OMD risk with travel-times, another significant trend in high-income countries is the increase in planned home deliveries [25–27]. However, planned home deliveries are rare in France, and this practice is even discouraged for single and low-risk pregnancies. It is therefore unlikely that these caused the recorded increase in the rate of OMD.

Another potential factor for OMD was highlighted by the French OMD observatory data. A recent study revealed that 23.3% of women had consulted an obstetrician in the 24 hours before OMD [28], and 6.8% of these consultations were within 6 hours. At the same time, we found a slight increase in the rate of antenatal hospitalizations for women living more than 45 km away from a maternity unit, which may be a result of the assumption of increased risk of OMD. However, hospital restructuring is in most cases accompanied by a reduction in the number of beds. This decrease, combined with an increase in the number of antenatal hospitalizations and non-programmable admissions due to the random onset of deliveries, leads to an inability to provide adequate care. The number of authorized beds should take into account the volume of deliveries and the randomness of this type of admission [29].

Although the OMD are mostly eutocic [30], our study confirms the increase in risk of adverse outcomes in OMD, including maternal death, stillbirths or neonatal death, hypothermia, polycythemias and newborn hospitalization. The increase in risk for fetus and newborns is well known [6,31–33], Our results for hypothermia [2,3,33], polycythemia [2] and neonatal hospitalizations [33] are similar to those found in other studies. An increase in perinatal mortality was also reported by other studies [20,34], which seems consistent with the increase in risks of stillbirths and in-hospital neonatal deaths observed in the present study. However, the risk of maternal mortality has rarely been studied.

Furthermore, our results point out a lower risk of OMD in urban centers and their suburbs than in the other types of living environments, but a higher risk in deprived areas. These points are consistent with previous results [19,33] and highlight the need for improved perinatal care networks in remote and disadvantaged rural areas, where emergency teams are often the first practitioners on the scene for cases of OMD. For this reason, all types of practitioners and caregivers should be trained to cope with OMD, according to emergency guidelines [7,30,35–37], wherever they occur [38,39].

This study has several limitations. Firstly, PMSI data do not distinguish between planned home births and unexpected OMD, and our study probably included women transferred postpartum when their delivery had initially been planned at home. In this group could have been included patients choosing to deliver at home for which complications occurred. Therefore, a small bias exists.

However, a French exposed vs. non-exposed cohort study [40,41], conducted by 47 midwives from 2009 to 2018, compared the outcomes of 1,192 planned at home deliveries to the outcomes of deliveries in maternity units. The study showed that only 0.3% of women who gave birth at home were transferred postnatally. In metropolitan France from 2012 to 2014, approximately 18,000 (0.8%) deliveries at ≥24 WG took place outside a maternity unit but with medical assistance (planned deliveries or emergency teams). From this cohort study, it can be estimated that 54 (0.3%) of these deliveries required postpartum transfer, which represents only 0.8% of the 6,733 OMDs included in our study, so that this risk of bias is very small.

Moreover, since planned at home deliveries are considered to concern very low risk pregnancies, this same cohort study showed that severe morbidity, particularly postpartum hemorrhage, was less frequent and less severe in the women who delivered at home than in controls who delivered in hospital. The same is true for neonatal morbidity.

Secondly, the French hospital discharge database is not a specific medical register. Thus, another limitation linked to the source of data concerns parity. Primiparous/multiparous status is not available in our data for OMD. We could not therefore adjust our results on this individual characteristic, which known for influencing the risk of OMD. However, a recent study assessing the metrological quality of hospital discharge data (PMSI) for perinatal indicators showed the reliability of the data used in our study [10], and another appraised the quality of hospital discharge data to identify maternal morbidity [42] and mortality [43].

It should be noted, however, that our data only includes hospital mortality since deaths that occur at home are not recorded in the PMSI.

Finally, our main strength is that we were able to estimate the frequency of out-of-maternity deliveries thanks to the PMSI database including all deliveries in France. We are also able to assess their impact on maternal mortality and on neonatal morbidity and mortality thanks to the linkage of information regarding the mother and her newborn.

These results confirm the findings of previous studies demonstrating that the PMSI can be used as a tool for obstetric and pediatric planning [44,45] because it allows units to be scaled according to the characteristics of the population served (individual characteristics and spatial distribution) [46], patient turnover in the context of regionalization and differentiation of care [47], the length of stay, and the random nature of unscheduled deliveries [47–49].

The use of the PMSI and these planning methods, in combination with analyses of the spatial distribution of the population's needs, would allow establishments to be sized appropriately and their geographical distribution optimized, thus limiting the risk of inadequate care and OMD. This type of planning based on the needs of the population and seeking to minimize risks is at odds with the current trend towards hospital restructuring, the objective of which is to reduce costs through economies of scale. In addition to the costs of restructured hospital units (obstetrics and neonatology), there are also the costs of treating OMDs in emergency departments and the hospital costs generated by the increase in obstetric and neonatal complications associated with travel times. If these costs are added to those of the restructured hospital services, it is not certain that an approach based on maternity closures and mergers will generate savings and be more efficient than planning based on the actual needs of the population. This line of thinking is especially relevant considering the lack of data on the medium- and long-term outcomes of children hospitalized following OMD, in particular their medical needs.

## Conclusion

Our results show that OMD have an impact on perinatal health outcomes, whether they are due to increased distance to the nearest maternity hospital or inadequate care. In future, these findings, which are based on validated PMSI data, should be considered carefully by the relevant authorities during planning and before any decisions are made to close or merge existing maternity units.

## Supporting information

S1 Table Supplementary file, risk factors for adverse outcomes: Sensitivity analyses (SA). (DOCX)
