# Supplementary material for: Out-of-maternity deliveries in France: A nationwide population-based study
Source: PLoS One. 2020 Feb 24;15(2):e0228785. doi: 10.1371/journal.pone.0228785 (PMC7039464; doi:10.1371/journal.pone.0228785)
Supplement: S1 Table — (DOCX) [file pone.0228785.s001.docx]

**S1 Table. Supplementary file, risk factors for adverse outcomes: sensitivity analyses (SA)**

|  |  | **Single babies born alive**  **N=1,999,453**  **(*Babies born before arrival*)** | | | |
| --- | --- | --- | --- | --- | --- |
|  |  | **Neonatal Death**  **(D0-D27)** | **Neonatal Hospitalization** | **Newborn Hypothermia** | **Neonatal Polycythemia** |
| Unexpected out -of-hospital deliveries |  |  |  |  |  |
| No |  | Reference | Reference | Reference | Reference |
| Yes |  | 1.9 (1.2-3.1) | 1.2 (1.1-1.3) | 5.9 (5.2-6.6) | 4.8 (3.5-6.4) |
| SA: multiple imputation |  | 1.7 (1.5-2.0) | 1.2 (1.1-1.3) | 6.1 (5.7-6.5) | 4.6 (4.0-5.1) |
| SA: maximum bias |  | 1.7 (1.0-2.7) | 1.1 (1.0-1.1) | 5.2 (4.6-5.9) | 4.2 (3.2-5.7) |
